# Epigenetic citizenship and political claims-making: the ethics of molecularizing structural racism

**DOI:** 10.1057/s41292-022-00286-4

**Published:** 2022-10-19

**Authors:** Jessica P. Cerdeña

**Affiliations:** 1 Department of Anthropology, Yale University, 10 Sachem Street, New Haven, CT 06511 USA; 2 Yale School of Medicine, New Haven, CT USA

**Keywords:** Epigenetics, Biological citizenship, Genetic citizenship, Epigenetic citizenship, Health disparities, Bioethics, Racism

## Abstract

Epigenetics has generated excitement over its potential to inform health disparities research by capturing the molecular signatures of social experiences. This paper highlights the concerns implied by these expectations of epigenetics research and discusses the possible ramifications of ‘molecularizing’ the forms of social suffering currently examined in epigenetics studies. Researchers working with oppressed populations—particularly racially marginalized groups—should further anticipate how their results might be interpreted to avoid fueling prejudiced claims of biological essentialism. Introducing the concept of ‘epigenetic citizenship,’ this paper considers the ways environmentally responsive methylation cues may be used in direct-to-consumer testing, healthcare, and biopolitical interactions. The conclusion addresses the future of social epigenetics research and the utility of an epigenetic citizenship framework.

The website for Chronomics a company that provides direct-to-consumer (DTC) testing for viral antigens and antibodies as well as epigenetic markers features the slogan “Making the unseen actionable.” The company implicitly promises consumers increased understanding of their biologies to spur change. Though the brief banner does not specify an aim toward individual, behavioral improvements or political claims-making, the message is one of empowerment: know your epigenetics and respond accordingly.

In 2020, bioethicist Charles Dupras published a commentary in *Nature Genetics* asking whether we are ready for DTC epigenetic testing. He called into question the legitimacy of epigenetic ‘evidence’ of lifetime exposures to stress or tobacco, imagining their use in forensic investigations and asylum evaluations (Dupras et al. 2020). Elsewhere, Dupras argues that emphasis on molecular insult is more likely to generate biomedical interventions to reverse epigenetic variation rather than prompting necessary policy and public health reforms (Dupras and Ravitsky 2016a). He invokes the possibility of discrimination based on epigenetic signaling and questions the moral responsibilities surrounding epigenetics, and whether parents and nation-states should be held accountable for the epigenetic programming of their children and citizens (Dupras et al. 2019, 2018; Dupras and Ravitsky 2016b).

This paper draws from insights gleaned through the design of a bioethnographic project on intergenerational trauma incorporating epigenetic measures of DNA methylation and neuroendocrine assays alongside anthropological methods of semi-structured interviews.^1^ I discuss the repercussions of seeking molecular ‘validation’ for the forms of social suffering currently examined in epigenetics research, including racism, trauma, poverty, and deprivation. I summarize science studies critiques of epigenetics research and its potential to mischaracterize the biological inscriptions of social experiences. Following Dupras, I further anticipate toxic interpretations of findings generated through social epigenetics research that might reproduce racialized claims of (epi)genetic determinism and buttress enduring support for biological essentialism. I advocate for cautious presentation of results and forethought over how these might be used by the public. Finally, building on the concepts of “biological citizenship” (Petryna 2013) and “genetic citizenship” (Heath et al. 2007), I develop the idea of ‘epigenetic citizenship,’ referring to how the molecularization of social suffering further constitutes the bodies of disenfranchised communities within the domain of biopolitical activism and intervention, and further propose scenarios by which epigenetic citizenship may manifest in the future.

## The promise and pitfalls of epigenetic inquiry

Eulalia (a pseudonym) was fifty-seven, separated, with one daughter. Originally from Mexico, she had been living in the states for the past twenty years—first in Long Island and now in New Haven. She was referred for psychoeducation after screening positive for depression in her medical visit, where her providers at our student-run free clinic were treating her for a host of issues, including back and knee pain, headaches, and bladder prolapse. Eulalia’s initial PHQ-9—a depression screening measure—was 17, suggesting moderately severe depression. Together, we were working to help her understand and manage her stress, particularly as it related to her experience as an immigrant. I traced my pen over the bulleted notes I had made during our last session on stress and migration.

- *migrated with ex-husband in search of work opportunities*
  - *ex-husband unfaithful, manipulative —* > *separated*
- *new spouse U.S. Citizen, controlled finances*
  - *daughter witnessed abuse*
- *priest told her to protect daughter, seek fresh start*
- *daughter withdrawn, failing*
  - *attempted suicide with medication overdose*
  - *now living with father*
  - *believes trauma from husbands’ abuse pushed daughter to suicidal thoughts*

My pen rested on that last line.

About forty minutes after our scheduled appointment time, Eulalia arrived: on seeing me, the creases around her eyes and the corners of her mouth split into a smile. She removed her hat, uncovering smooth black hair with a few rebellious grays springing from her temples. I led her to the clinic room and we each took a seat.

I slid my fingers across my white presentation binder and turned to the section on social support. Before I could finish reading through the content of the educational module, Eulalia interrupted me.

“My daughter is everything to me. She and I only have each other. But she wants to leave to go back to live with her father in Long Island. She thinks things will be better there. I am so afraid for her.”

Hot tears streamed down Eulalia’s face.

“She tried to kill herself,” she stammered. “She took all the pills out of the medicine cabinet and tried to take them. I found her when she was starting on the second bottle. We took her to the hospital, but we told them it was an accident, that she’d misread the label. Praise God, she was okay. But I am so afraid for her. I think this is all my fault.”

Eulalia’s story pierced me, shaping the way I conducted future psychoeducation sessions with migrant patients experiencing depression. As I gently interrogated about their relationships with their children, I learned more and more about how these individuals—particularly migrant mothers—feared their traumas affected their children. Whether immigration detention, financial stress, child sexual abuse, or intimate partner violence rose to the top of their list of stressors, many of these women felt that their children’s emotional or psychological conditions resulted in part from their mother’s wounds.

I was not familiar with the literature on intergenerational trauma at the time I conducted these sessions with Eulalia and other women like her. However, my MD/PhD training led me to a recent article that seemed to address what I had observed.

The study, led by Dr. Rachel Yehuda, a professor of psychiatry and neuroscience at the Mount Sinai School of Medicine, discussed associations of DNA methylation—a form of epigenetic modification—at a gene involved in the stress response among parents who had been exposed to the Holocaust prior to having children, and the children themselves. In other words, the study demonstrated—albeit weakly—a biological embedding of parental trauma on the epigenomes of their offspring.

This potential for biological underpinnings of intergenerational trauma drove me to further research, and supersized the index for questions I had developed. By what mechanisms could parental (specifically maternal) trauma influence offspring? How do mother’s narratives of trauma correspond to epigenetic signals? Do epigenetic modifications at stress-related genes affect the stress response? My research aimed answer these questions using a bioethnographic approach.

As I began to share my ambitions with colleagues—particularly my Black and Latinx peers—many shared their hesitations. They made comments like, “We’re not broken,” or “Don’t you think that White supremacists would have a field day with some of this research?” Those exchanges left me with lead in my stomach. I had hoped my research would challenge neoliberal and medicalized impulses to blame women like Eulalia for ‘poor parenting’ rather than considering how experiences of structural vulnerability might shape neuroendocrine programming, predisposing oppressed families to psychopathology (Cerdeña et al. 2021). I had not imagined that my yet-unrealized work might be weaponized in ways that could reinforce social inequality. This paper interrogates these tensions and further considers how social epigenomics may reshape self-regulation, identity, and relationships between individuals and systems of biopolitical governance.

## The limitations of molecularizing social suffering

On May 27, 2016, the *Atlanta Black Star* posted a Facebook video with the caption: “Slavery Happened A Long Time Ago? Scientist Are Now Saying the Trauma May Be Encoded In The Genes of Black People.” In it, psychiatrist and neuroscientist Dr. Rachel Yehuda discusses her paper on intergenerational transmission of epigenetic marks in children born to survivors of the Holocaust (Yehuda et al. 2016). The makers of the video extend Yehuda’s findings to racialized trauma, suggesting that the mental and physical anguish endured by Africans who were captured during the transatlantic slave trade may be “encoded in the genes” of their descendants. To comment on this phenomenon, the video turns to Joy DeGruy, a social work scholar and author of *Post-Traumatic Slave Syndrome*. DeGruy argues that it is “not plausible” for Black Americans to have averted stress-related illness due to the multiple traumas the generations of their ancestors sustained over hundreds of years. As of July 2021, this video has been viewed more than 4.7 million times.

Viewer responses reflect widespread feelings of affirmation.^2^ One top commenter on the video notes, “I knew this before I [*sic*] was even scientifically know [*sic*] by looking at the hurt and anger in my mothers and elders.” Another insists, “black ppl [people] did not need science to prove that if a people are made to be mentally, physically, spiritually, financially and socially oppressed …while tortured and tamed, the effects thereof will essentially carry on down through their bloodline.” For these viewers, molecular evidence of the trauma of slavery is redundant and unnecessary: the narratives and embodied experiences of their relatives and ancestors reveal more than could any chemical tags on DNA.

However, many members of the academic community have received the early findings of social epigenetics research with greater enthusiasm. Scholars across fields of biomedicine, philosophy, sociology, and anthropology have acclaimed the potential for epigenetics to characterize the biological impact of social forces (Chung et al. 2016; Kuzawa and Sweet 2009; Non and Thayer 2015; Sullivan 2013; Thayer and Kuzawa 2011). In particular, biological anthropologists Zaneta Thayer and Chris Kuzawa emphasized the promise of epigenetics to reveal how factors like social and economic inequality “get under the skin” to create health disparities (Thayer and Kuzawa 2011). Calling it a “science of social science,” medical physicist Emma Chung and her cross-disciplinary team of social science and biomedical scholars claim epigenetics can transform social science by forging a “social epigenome” that encompasses the “myriad miniscule interactions that are at once socioculturally and materially, relationally and biologically situated” (Chung et al. 2016). These commentaries reflect a keen, transdisciplinary interest in novel opportunities for epigenetics to bridge the life and social sciences and to translate into interpretable information such methodologically elusive phenomena as racism, trauma, and poverty.

Seeking to enrich the bank of data characterizing the “social epigenome,” the National Institute for Minority Health and Health Disparities (NIMHD) and the National Cancer Institute (NCI) created a funding opportunity in 2016 dedicated to “Social Epigenomics Research Focused on Minority Health and Health Disparities.” In the funding announcement, the Institutes state their intent to support investigations that identify and describe the mechanisms that influence gene function in a way that modifies health risks in minority populations. The announcement elaborates further in its Research Objectives:

The overarching objectives of this initiative are to (1) advance the science of epigenomics focused on minority health and health disparities, (2) expand approaches for understanding epigenetic mechanisms by which social factors lead to biological changes that affect health disparities, and (3) promote epigenetics research to better diagnose disease risk or resiliency among disadvantaged populations. Successful projects will support human-based epigenomic research, with a particular focus on the identification and study of human epigenetic marks that are of social origin or are substantially influenced at a population level by social processes.

The National Institutes of Health (NIH) renewed the funding announcement in 2019, and it has remained open in years since. Through these grant opportunities, the NIH, emblematic of the scientific ‘mainstream,’ endorses fitting epigenomics research as a missing link between social factors and inequitable health outcomes. No longer a fringe idea advanced by a contingent of socially conscious scientists, “social epigenomics” has gained traction with the world’s largest funder of health research (Viergever and Hendriks 2016).

The academic and lay person responses to the promises of epigenetics remain out of sync. The research community believes that “social epigenetics/epigenomics,” that is, the study of environmentally sensitive molecular modifications to the genome that correspond to social processes, hold the key to understanding the biological underpinnings of health disparities affecting socially disadvantaged communities, particularly racially oppressed groups. By contrast, members of these communities express a belief that epigenetic evidence merely corroborates what might already be understood from listening to their family and life histories (Nelson 2016). In between the two, science studies scholars have urged caution regarding the risks of social epigenetics to contract complex and dynamic social environments and to individualize structural and political forces (Louvel 2020; Saldaña-Tejeda 2018).

Proponents of social epigenetics research would like the epigenome to behave like wet clay with the footprints of adversity impressed upon it, waiting to be discovered by a careful investigator. The reality, however, is much more complicated, hindered by methodological issues and limitations to data availability (Non 2021). In this section, I review the current limitations of epigenetics research and discuss their implications for examining social variables.

First, epigenetic signals are meager and difficult to detect. CpG methylation, or the attachment of a methyl (–CH_3_) group to a cytosine residue at a region enriched in cytosine and guanine nucleotides, is a commonly measured epigenetic mark. Researchers transform the DNA to distinguish methylated cytosine residues from unmethylated cytosines, through a process known as bisulfite conversion, and then amplify the DNA. The most popular methylation assay,^3^ Infinium by Illumina®, then uses a pair of probes, one for unmethylated residues and another for methylated residues, to tag the nucleotides on these amplified sequences. A reader can then measure the intensity issued from these probes and calculate the ratio of the intensity from the methylated probe to the total intensity of the methylated and unmethylated probes (Du et al. 2010). The results, called beta values, can range from 0 (unmethylated) to 1 (fully methylated). These may also be reported as methylation percentages.

However, a meaningful difference in beta values, or methylation percentages, is often very small. For instance, Perroud et al. (2011) reported a significant difference in methylation levels at the glucocorticoid receptor, *NR3C1*, with history of sexual abuse in patients with bipolar disorder: those without a history of sexual abuse had methylation levels of 0.128 (s.d. 0.02), whereas those who had experienced sexual abuse had methylation levels of 0.141 (s.d. 0.02) (Perroud et al. 2011). The difference between these is just 9%, a statistically significant result (*P* = 0.011) of uncertain biological consequence. This raises concerns over interpretations of methylation data, especially from individual loci with few CpG sites; associating methylation levels with intangible experiences further complicates the field of social epigenetics.

Additional methodological challenges arise in the laboratory during sample processing. As genetic anthropologist Amy L. Non notes, microarrays—the ‘chips’ or plates on which most epigenetic analyses are conducted—are subject to extreme batch effects, or wide variation in results depending on the sample’s location on the chip and the technician who handled it. These batch effects limit opportunities for pooling samples across studies or time points, which would allow for larger sample sizes. As such, social epigenetics studies often focus on small samples and test candidate genes, rather than the entire epigenome, in order to achieve adequate statistical power. Findings in targeted studies of candidate genes have not been consistently replicated in epigenome-wide studies (Non 2021). Epigenetic research often relies on convenience samples that do not reflect racial, ethnic, and geographic diversity of the populations they intend to represent (Evans et al. 2021).

Furthermore, the tissue samples most accessed in epigenetics research may not be appropriate to analyze the questions of social epigenetics. The physiological systems implicated in social processes such as trauma, discrimination, and chronic stress are neuroendocrine and neuropsychiatric, and are likely to be best examined by measuring methylation in brain tissue. However, given the inaccessibility of brain tissue in living humans, researchers must settle for less invasive samples like blood, saliva, and buccal (i.e., cheek) cells. Although methylation patterns in saliva have been shown to be consistent with those in brain tissue, potentially offering an appropriate substitute for researchers interested in the epigenetics of psychosocial schema, saliva introduces issues of cellular heterogeneity due to the presence of both leukocytes and buccal cells (Smith et al. 2015). This heterogeneity may potentially dampen statistical signals if researchers do not properly account for it.

Another shortcoming of current epigenetics studies is their failure to account for the interactions between genotype and epigenotype; most focus on one or the other. Although humans share 99.9% of their DNA with one another, those key polymorphisms may predict differences in our responses to the environment and may be crucial to distinguishing the role of epigenetics in mediating health disparities. Yet very few studies to date have included both analyses of relevant DNA polymorphisms and methylation. One study of the monoamine oxidase gene, *MAOA*, which has been implicated in multiple mental disorders, examined mRNA expression imbalances between two single nucleotide polymorphisms (SNPs), or sites of known DNA variation, measured methylation in the gene promoter, and genotyped subjects for a repeat polymorphism in the promoter (Pinsonneault et al. 2006). The authors used postmortem brain tissue samples from individuals diagnosed with bipolar disorder or schizophrenia, and healthy controls. They found that methylation highly correlated with mRNA expression imbalances and that polymorphism genotypes strongly associated with a haplotype block between the promoter and the terminal marker SNP (Pinsonneault et al. 2006). Their results imply functional interactions between genetic scaffolding, epigenetic tone, and health. Failing to include relevant genomic information can result in an incomplete rendering of the impact of social environmental on human health.

Some more cautious researchers may be reluctant to genotype participants for risk of attributing too much of their embedded biologies to genes; however, excluding this data point limits the validity of epigenetics as a psychosocial biomarker. Given that DNA methylation influences gene expression, knowledge of any underlying allelic variation can inform understandings of the role of epigenetics in clinical phenotypes.

Understandably, the risk of encouraging genetic determinism is acutely felt; concerns over associating adverse experiences with genotypes are especially salient in the era of CRISPR/Cas9 and approval by the U.S. National Academies of Sciences and Medicine to edit human embryos (National Academies of Sciences 2017). For this reason, scientists should exercise extreme caution when conducting social epigenetics research and disseminating their findings. I return to this issue in the next section of this paper.

Perhaps the most pressing issue for both social scientists and prospective social epigeneticists is the lack of instruments that accurately quantify the impacts of adversity. Without effective tools to operationalize social suffering, social epigenetics will lack the methodological rigor necessary to fulfill the lofty expectations academics have set for it. Consider discrimination as an example: although social scientists understand that it impacts health, it must be measured indirectly. Social epidemiologist Nancy Krieger comments that this may be achieved by comparing the observable risk factors between dominant and subordinate groups that result from discrimination. These include self-reported, individual experiences of threat and discourtesy (Williams 1997), as well as structural measures of segregation, poverty, housing quality, population density, and toxic exposures (Krieger 2000; Krieger et al. 2016). However, there is no consensus on how to comprehensively measure discrimination across multiple dimensions (Williams and Mohammed 2009). This makes it difficult, if not impossible, to properly evaluate epigenetic changes in response to a discriminatory environment. Further, a recent systematic review of epigenetic research found that experiences tend to be individualized, likely due to measurement ease, rather than conceptualized according to social, economic, and political stratifications that may more appropriately reflect gradations of oppression (Evans et al. 2021). This also limits the timescale of the ‘environment’ to a single cross-section, rather than as the embodiment of cumulative experiences from the past as well as the present (Lamoreaux 2016).

Given the knottiness of these interactions, it is worth asking whether it is useful to operationalize them at all. Berlin-based anthropologist Jörg Niewöhner critiques the structuralist, “pragmatic reductionism” of social inequalities in laboratory-based research (2011). Commenting on how molecular biologists are beginning to shift their gaze to adverse early life events, Niewöhner warns against the “molecularization of biographies and milieu” (2011, p. 291). He describes this ‘molecularization’ as “the extraction of significant events from people’s biographies, from particular and situated socio-cultural histories and from their embeddedness in particular milieu and everyday lives, and their conversion into standardized representations of particular forms of social change that can be correlated with the material body” (Niewöhner 2011, p. 291). Yet, Niewöhner notes that the interconnections between ‘nature’ and ‘culture’ may render plausible the dependence of something as “seemingly hard-wired” as gene expression on local meanings (Niewöhner 2011, p. 293). Anthropologist Margaret Lock’s analyses of epigenetics echo this sentiment. Lock notes that histories, social worlds, politics, and subjectivities are inextricable from material bodies and deserve full recognition (Lock 2015). She problematizes biologists’ need to think linearly and advocates for “overlapping,” “interrelated,” and “stochastic” conceptualizations to avoid “systematized reductionism” (Lock 2015, p. 262). Further, science and technology studies scholar Maurizio Meloni examines how social epigenetics disrupts conventional understandings of “natural” or “genetic” inequalities and “social” inequalities and its implications for theories of justice (2015). These commentaries pose additional obstacles for scientists aiming to conduct meaningful social epigenetics research: they reveal the importance of qualitative data—ideally thick, ethnographic narrative—to contextualize and give meaning to epigenetic findings and the challenges inherent in data interpretation.

The necessity of ethnography to interrogate the subjectivities of social suffering is evident to anthropologists and sociologists, but less intuitive to molecular biologists and geneticists. “Social suffering” refers to the trauma, pain, and disorders that result from the imposition of political, economic, and institutional power (Kleinman et al. 1997). In other words, it is the suffering caused by social forces, whether global markets or interpersonal relationships. Medical anthropologist Arthur Kleinman emphasizes the primacy of a suffering individual’s local world, specifically their local moral world, in which their actions and beliefs are shaped by cultural, political, institutional, and social feedback (Kleinman 1992, 1997, 2006). Trained ethnographers can characterize the structure of this local moral world and examine how individuals’ experience of it is reflected in their inner affective states, or subjectivity (Biehl et al. 2007). Analyses of these particularities, the sparks generated from institutional, political, and intersubjective frictions, are critical to our current understanding of social suffering. The compaction that occurs using quantitative instruments universalizes social experiences, presuming all people internalize and interpret events in the same way. Inasmuch as social epigenetics research aims to examine the biological signatures of social suffering, identifying a way to meaningfully integrate ethnographic data with operationalized measures of social experience and methylation levels will remain a challenge.

Medical anthropologist Elizabeth Roberts proposes an integration of the social and life sciences through bioethnography, which combines biological and ethnographic data to enrich understandings of health inequalities (Roberts and Sanz 2018). Rather than merely presenting these data in parallel, preliminary investigations that situate each methodology as valid forms of knowledge production can inform research questions and further data collection to produce “better numbers” (Roberts 2021). Bioethnography offers an opportunity to empirically evaluate how developmentally plastic biological systems (e.g., epigenetic, hormonal, neuronal) respond to local ecologies, resulting in differentiation even within socially defined groups (Lock et al. 2021).

In my own research, I have engaged ethnographic inquiry to inform model predictors for embodied expressions or biomarker. As an example, thematic coding of an interview section on adversity can produce categorical variables (e.g., “adversity is part of life,” “I have suffered too much, it is unfair,” “I always look at the positive side of a difficult situation”) that can be entered into regression models to predict telomere length. As such, ethnographic data can advance knowledge of the interplay between “local” or “exposed” biologies and their constitutive environments (Lock 1993; Wahlberg 2018).

### (Epi)genetic determinism

Research in social epigenetics provides an opportunity to recontour the invented boundaries between the ‘biological’ and the ‘social’ by imagining pathways of potential in which genes and environments exist in dynamic interplay (Richardson 2017; Waggoner and Uller 2015). Epigenetics as a field, however, continues to insist on the primacy of the gene: the environment exists only to the extent that it can be measured as methylation, phosphorylation, acetylation, or other molecular change to chromosomal DNA (Seeberg et al. 2020). Further, molecular targets lead to molecular treatments, lures for the multibillion-dollar pharmaceutical industry. Whereas multidimensional, historically situated, and socio-politically contingent conceptions of the environment require costly policy interventions that run contrary to a neoliberal ethos, molecularizations of the social environment promise lucrative earnings through drug development (Krieger 2005; Seeberg et al. 2020). These misplaced incentives drive epigenetic determinism.

Briefly returning to the Atlanta Black Star video, I want to highlight a different set of user responses. These, I found on YouTube, where the video had been posted on the same day. One commenter asked, “Do [*sic*] they find the DNA marker for low IQ yet?” advancing a racist and scientifically flawed idea that Black people have broadly lower intelligence. Another said, “This may be propaganda to put all [B]lack people in mental internment camps” (Atlanta Black Star 2016). This troubling suggestion leads me into my next sections on (epi)genetic determinism and ‘epigenetic citizenship.’

The idea that social exposures may be evident on a molecular level—and further, that these molecular marks are heritable—may rationalize beliefs in the inferiority of communities who disproportionately suffer. Put another way, people may ignore the role of the social and structural forces that mediate epigenetic change and instead view any epigenetically associated health deficiencies as inherent to the population. The YouTube commenter who proposed the forcible placement of Black people into “mental internment camps” focused on these molecular imprints and their implications for mental health and social functioning, rather than considering solutions that would remedy the sources of trauma that induced these epigenetic changes.

This disturbing set of beliefs harkens to the origins of behavior genetics, or the field bridging psychology and evolutionary biology that studies genetic and environmental influences on human behavior, the ‘nature/nurture divide.’ Sociologist Aaron Panofsky highlights how behavior genetics has defined itself as a discipline through controversies, ranging from racial achievement gaps to criminality to gayness (2014). For instance, early theories on behavioral genetics proposing that human behaviors including sexual assault, xenophobic violence, and gendered, heteronormative divisions of labor may reflect selective advantages prompted accusations of scientific racism and biological essentialism (Panofsky 2014; Wilson 1978, 1975). Also famously, psychologist J. Philippe Rushton characterized racial hierarchies in intelligence and social behavior that corresponded to grotesque morphological characteristics, including brain and penis size (1995). Rushton’s work informed the longstanding *New York Times* bestseller, *The Bell Curve*, which argued for genetically determined differences in intelligence between Black and White populations and further popularized tacit understandings of racial essentialism that abet structural racism (Kaufman 2014). These iterations of racist science derive from the convergence of European colonization and Enlightenment philosophy at which point the ‘empiricism’ of the latter rationalized the brutalism of the former (Cerdeña 2021).

Social epigenetics research, if not carefully executed and presented, may encourage interpretations that reinscribe racial essentialism (Lappé and Landecker 2015; Lloyd and Müller 2018; Meloni and Testa 2014; Roberts and Rollins 2020). Homing in on outcomes, and their proximate epigenetic modifiers, leads to the conclusion that health disparities emerge at the level of individuals, or populations of like individuals, rather than because of shared environmental exposures. This thinking is exemplified in the YouTube comment about “mental internment camps:” the user immediately thought to restrain the group of people who may be prone to mental illness due to changes in their DNA, rather than proposing reparations to alleviate the historical trauma that hypothetically induced those changes. The user focused on the most tangible findings—those that influenced, or affirmed, their perceptions of Black people—and sought to intervene on those.

This example demonstrates why the risk of (epi)genetic determinism is especially high for intergenerational studies (Waggoner and Uller 2015): in these cases, the molecularized bodies under examination are one degree removed from the social processes that shaped them. Black people today presumably have not experienced the traumas associated with slavery, yet the Atlanta Black Star video suggests they may still be predisposed to post-traumatic stress disorder due to an inherited tag in their epigenomes. Due to the presence of this mark, they are ‘determined’ at birth to have an elevated risk of mental illness. Although this statement is accurate, it overlooks the inextricable role of the social adversities that effected the epigenetic change in the first place. Unintended characterizations of epigenetic marks as permanent and persistent among survivors of adverse social environments—and their descendants—risk legitimating essentialist ideas of inherent flaws in oppressed populations, particularly racialized groups (Lloyd and Müller 2018). Limited public understanding of epigenetic mechanisms—particularly the potential for differential methylation to shift or resolve over time (Simpkin et al. 2015)—relative to common knowledge of genetics and heritability lend themselves to difficulties distinguishing inter- and transgenerational epigenetic findings from genetic risk (Dubois and Guaspare 2020).

It is incumbent upon researchers in social epigenetics to ensure that phenotypes associated with epigenetic change remain embedded within environmental milieu. Researchers should take care to avoid interpretations that yield headlines like “Is Trauma Genetic?” (Shulevitz 2014) by limiting their use of terms like “heritable” and “genetic” in abstracts and conclusion sections. Researchers should also take care to respond to media requests promptly and to ensure that only authors with sensitivity to these ethical concerns interact with media outlets. By curating research findings as best they can, scientists in social epigenetics can prevent the emergence of interpretations that may harm their study populations.

## Epigenetic citizenship

In this section, I explore the liminal spaces of self-conception and construction of the environmentally embodied ‘citizen’ emerging from epigenetics research. I use the term ‘epigenetic citizenship’ to describe how individuals may increasingly consider their pliable, molecular selves in lifestyle and health decisions and to further consider how molecular data might be deployed to legitimate biopolitical claims or justify interventions.

Deborah Heath, Rayna Rapp, and Karen-Sue Taussig proposed the concept of “genetic citizenship” in response to advances in genomics and intensifying interest in the genetic explanations for human health, disease, and ways of being. Heath, Rapp, and Taussig observe that the process of “geneticization” mobilizes researchers, health activists, and public funding sources “as people learn to ‘think genetically,’ to see themselves in terms of genetic attributes and limits—or as investment possibilities” (2007). This ‘auto-geneticization’ engenders novel forms of identity and claims-making, prompting people to contemplate how their genetic selves valence understandings of illness, ability, and advocacy. The authors propose the term “genetic citizenship” to link “discussions of rights, recognitions, and responsibilities to intimate, fundamental concerns about heritable identities, differential embodiment, and an ethics of care” (Heath et al. 2007). Rooted in the discourse of genetic citizenship, ‘epigenetic citizenship’ examines the sociopolitical significance attached not to the fixed, coding sequences inherited by chance, but rather the flexible, chemical modification acquired through violence.

Epigenetic citizenship is performed at the interface between individuals and healthcare providers, insurance agencies, corporations, governments, and funding organizations; individuals may also enact epigenetic citizenship through self-regulation and behavior change. Epigenetic citizenship relies on Niewöhner’s notion of the “embedded body,” or a body permeated by its past and present social and material environments (Niewöhner 2011). Epigenetic citizenship advances this idea forward, proposing that increased recognition of the interdigitation between molecular body and environment makes way for new interactions between suffering individuals, researchers, and policymakers. As science historian Sarah S. Richardson explains, “Epigenetics does not so much ‘make plausible’ the embedded body; rather, it fixes the molecular gaze on the embedded body… and elevates it to the center of biomedical theory, intervention, and surveillance” (2015, p. 227).

As epigenetic testing is not yet widespread, iterations of epigenetic citizenship are largely imagined. Here, I discuss these articulations of epigenetic citizenship at the level of the individual—as a healthcare consumer and community member—and the state through case-based scenarios of self-regulation, identity, claims-making, and state control.

### Self-regulation

“We tell our pregnant patients all the time to quit smoking, but it doesn’t help,” my colleague, an addiction medicine fellow, tells me, “But if we told them, ‘We know of an epigenetic mark that comes from cigarette smoking that could be passed on to your unborn child,’ that might make the difference.”

After learning about my research, a colleague of mine proposed we collaborate on an intervention to prevent substance use among pregnant women. Their suggestion about using epigenetic science to influence behavioral health illustrates how patients might be transformed into epigenetic citizens.

Smoking has been shown to influence DNA methylation at cancer-causing genes (Belinsky et al. 2002); furthermore, children exposed to cigarette smoke during pregnancy exhibit significant methylation changes that may predispose them to disease later in life (Breton et al. 2009). Knowledge of these epigenetic associations, and the construction of epigenetic citizenship, molecularizes smoking behavior. Whereas current interventions on smoking focus on health consequences for the individual-and, to a lesser extent, social disruptions—the consciousness of an epigenetic citizen concerns the molecular bodies of both themselves and their children, especially if the individual is a woman.^4^ Epigenetic citizenship may thereby manifest through cessation of smoking or other substances (Wong et al. 2011), weight loss (Campión et al. 2009), and more attentive parenting (Weaver et al. 2004) as individuals seek to secure not only their own molecular and health futures, but also those of their children and future children. With each novel association between epigenetic marks and health outcomes, the scope of epigenetic citizenship will expand.

The enactment of epigenetic citizenship may arouse feelings of guilt and helplessness; individuals may feel responsible for their children’s health risks, or feel unable to change their circumstances, despite awareness of the ‘molecular damage’ it may cause. This may be an unintended consequence of social epigenetics research and its tendency toward interpretations that individualizing health inequalities (Romijn and Louvel 2021). For this reason, healthcare providers and biotechnology companies should exercise moral caution before obtaining, disclosing, or commodifying, epigenetic data.

### Identity and epigenetic selves

On its homepage, EpigenCare promises to “match products to your skin’s epigenetics” (EpigenCare, Inc. 2019). Muhdo encourages consumers to “discover [their] biological age and take control of [their] genetic health” and invites them to “reverse the ageing process” (Muhdo Health Ltd. 2022). TruMe offers customized wellness routines based on changes to DNA (TruMe 2020). These companies seek to commodify and market empowerment, or this imagined idea that greater technoscientific knowledge of individual biological variation—including of the epigenome—will enable a healthcare consumer to tailor their (pharmacoepigenetic) treatments and optimize their health (Chiapperino and Testa 2016; Mai and Altucci 2009). This bioeconomic venture capitalizes on human interest in seeking and increasing their value, including within their own bodies (Clarke et al. 2010).

Beyond this, knowledge of a person’s epigenetic self can lead to new characterizations and imaginations of community. Consider this exchange between two characters—Sam, a Black student activist, and Gabe, her White boyfriend—from an episode of the political Netflix series, *Dear White People*. In the scene, Gabe prompts Sam, who is biracial and continually grapples with feelings of belonging in racially segregated spaces, to share her experiences of racism more vulnerably. Sam snaps back, “Have you heard of epigenetics?… it’s the inheritance of pain. Basically, scientists have figured out that people who experience intense trauma, like slavery, pass that down through their DNA, so pain and suffering is literally in my blood” (Holden 2021). At once, Sam affirms that she does not owe Gabe access to her distress, but also cements her identity as a Black woman, epigenetically linked to common enslaved ancestors. She imagines an epigenetic self that ratifies her American Blackness, her ancestral ties to enslaved Africans.

This demonstrates the concept advanced by anthropologist Paul Rabinow of “biosociality,” or the formation of individual social or group identity based on biogenetic stratification and risk (1996). Epigenetic citizenship emerges through the relational construction of self and community according to shared epigenetic marks.

This more intimate, intersubjective expression of epigenetic citizenship can range from superficial, such as a biosocial identity based on an epigenetic skin type, to the profound, as with a biosocial identity as excessively aged at a cellular level due to racial weathering (Geronimus et al. 2006). Epigenetic selfhood attains particular salience in situations of assisted reproduction, in which individuals born through gestational surrogacy may reimagine their bio(epi)genetic kinship, race, and socioemotional histories based on the purported influence of their alternate intrauterine environments (Keaney 2021; van Wichelen 2022).

This further ties into epigenetic identification with mass historical traumas. Perhaps a methylation tag at a gene involved in metabolism might unify survivors of the Chinese Cultural Revolution or the Cambodian genocide, who overcame starvation and forced labor (Shen et al. 2019; Zimmet et al. 2018). A signal at a stress-related gene might tether Americans with traumatic memories of the 9/11 terrorist attacks (Uddin et al. 2018). Modifications at genes involved in parental attachment might lend themselves to biosocial communities among adults who had been abandoned at Romanian orphanages in the late twentieth century (Esposito et al. 2016; Non et al. 2016). Mothers and the children they bore during the COVID-19 pandemic may find solidarity worldwide through shared molecular marks (Provenzi et al. 2020). As DTC epigenetic testing increases in popularity, these novel articulations of epigenetic citizenship may become more common and shape political formations and claims.

### Claims-making

In 2018, the California Legislative Assembly passed a resolution encouraging awareness of the impact of intergenerational trauma identified through epigenetic study on certain California citizens. The resolution acknowledges that “our genes carry extreme evidence of trauma experienced by our ancestors,” specifically citing the genocide and forced relocation of Native Americans to residential boarding schools and the enslavement of African Americans. The author of the resolution, Representative Reginald Jones-Sawyer, a Black man who serves a predominantly Black and Latinx district, mentions “molecular scares adhering to our DNA” and how “DNA holds the traumatic history” of ancestors (2018). Although the bill does not explicitly invite citizens to seek reparations for the atrocities committed by the U.S. government, it acknowledges the molecular and psychological harm of these acts. The legislature implicates epigenetics in triggering intergenerational health consequences across generations, stating “scientific research suggests that the negative effects of trauma can be inherited and parents may actually transfer the consequences of experiencing intense psychological trauma to their children via an epigenetic process” (Jones-Sawyer 2018).

In this section, I posit the deployment of epigenetic citizenship in cases of environmental risk. Residents of communities contaminated by hazardous waste, toxins, and air pollutants are disproportionately likely to be poor and minority (Bullard 2000). The environmental justice movement emerged to help affected communities surmount the structural barriers erected against them so they could issue grievances and receive restitution (Čapek 1993; Cutter 1995). Environmental justice activities involve claims-making (Best 1987) and advocating for the equitable distribution of environmental risk. Within claims-making interactions, the incorporation of epigenetic data may provide additional, invisible evidence that favors sufferers.

Epigenetic mechanisms have been hypothesized to play a role in the pathogenesis of disease conditions associated with environmental pollutants (Hou et al. 2012), and heavy metals in particular have been shown to cause epigenetic alterations (Baccarelli and Bollati 2009). To date, studies in humans have been limited; however, preliminary findings confirm in vitro results (Li et al. 2013; Sharavanan et al. 2020). These epigenetic changes are significant as they may precipitate the development of cancer or various psychiatric and neurocognitive disorders (Hou et al. 2012).

Consider the well-known example of lead contamination of the water supply in Flint, Michigan. The cutoff for lead poisoning in children is a blood lead level of 5 μg/dL, at which point the child is targeted for clinical intervention (Taylor et al. 2016). Due to the developmental consequences of lead poisoning, children with lead levels at or above the reference level are often eligible for services including nutritional interventions, educational assistance, physical and behavioral therapy, and other medical services. However, the 5 μg/dL reference value is based on the top 2.5% of child blood lead levels found in the National Health and Nutrition Examination Study (NHANES) and has no clinical significance (Centers for Disease Control and Prevention 2017), meaning children with levels below 5 μg/dL may still suffer long-term health consequences (Taylor et al. 2016). This poses challenges for families of exposed children whose lead levels fall below the cutoff: they fear their child’s developmental course may be permanently altered, yet they lack a formal right to the resources that might keep their child on track.

Here, epigenetic evidence of lead toxicity would strengthen claims to services for exposed children. Practicing epigenetic citizenship, parents of exposed children could employ methylation data to support demands against the state and against landlords or housing authorities. As individuals begin to understand themselves in epigenetic terms—becoming cognizant of the microscopic marks made by environmental insults, and the diseases these marks may engraft—they may distrust conventional diagnostic criteria, which do not transcribe their embedded bodies.

Sociologist Élodie Grossi describes the mobilization of transgenerational epigenetics research among activist communities advocating for reparations relating to chattel slavery in the United States (2020). As research on biosocial inheritance and transgenerational transmission of epigenetic marks advances (Bošković and Rando 2018; Heard and Martienssen 2014; Hoke and McDade 2014), the practice of epigenetic citizenship may extend beyond contemporary environmental exposures to historical wrongs.

### State control

The examples of behavioral health and environmental justice explore the performance of epigenetic citizenship by individuals, but epigenetic citizens may also be constructed through state and institutional exercise of biopower (Foucault 1990). I now probe the possibility of adopting epigenetic measures in public health surveillance and intervention, specifically to reduce the social and economic burden of mental illness and its comorbidities. I project the use of DNA methylation at neuroregulatory genes as biomarkers for psychiatric illness, which may then inform diagnoses and trace disease trajectories (Singh et al. 2011). To consider how this might inform public health, I apply this to a hypothetical scenario of epigenetically screening combat veterans to determine their predisposition for psychiatric disorder and triaging high-risk veterans to preventative mental health interventions.

Mental illness is a leading cause of death and disability in the United States (Kessler and Wang 2008). The cost of mental illness is vast: the global economic burden is estimated at $2.5 trillion, most of which are in indirect costs such as incarcerations, homelessness, and lost productivity (Kessler and Wang 2008; Trautmann et al. 2016). Veterans are especially vulnerable to mental illness, and many suffer from psychiatric and substance use disorders (Hankin et al. 1999; Watkins and Pincus 2011). In 2008, the Department of Veterans Affairs (VA) spent 2.7 times more on veterans with mental illness or substance use disorders relative to those without (Watkins and Pincus 2011). Interventions designed to reduce the psychiatric suffering of these veterans, and the resource-intensiveness of their care would be expedient for the U.S. government.

Epigenetic alterations have been associated with the development of mental illness in the wake of traumatic exposure (Mehta et al. 2013). *FKBP5*, the gene discussed in the Atlanta Black Star Video, has been proposed as a target for epigenetic alteration given its associations with post-traumatic stress disorder, suicidality, and bipolar disorder (Klengel et al. 2012; Zannas et al. 2016). A candidate-gene approach to quantify methylation currently costs around $45 per sample, and prices may decrease as technologies continue to improve. At limited expense, returning veterans might be screened for methylation changes that may predict psychiatric illness prior to the presentation of clinical symptoms. Although the implementation of this practice would only follow additional, direct evidence supporting its use as secondary prevention, the possibility fertilizes new territories of epigenetic citizenship. Taking precedence over ‘exposure’ surveys or subjective narratives of war trauma, methylation data elaborate the veteran’s molecular identity, directing state-sponsored biomedical intervention.

In discussing these hypothetical cases of epigenetic citizenship, I intend to highlight how this awareness of molecular embeddedness may achieve both productive and damaging ends. On the one hand, epigenetic embodiment may be used as a tool of empowerment, engaged in new articulations of health consciousness and scientific activism. One commenter on the Atlanta Black Star Facebook video took up this mantle, saying,

“Post traumatic slave syndrome. We never got any psychological help or any type of help for what we had endured in the past. America just want us to get over it foh [f***k outta here]. Its [*sic*] coded in our DNA. It's embedded in our genetic structure. We never really healed. Its [*sic*] time we start to heal each other. This is scientific proof that slavery still has an effect on us. So what's going to be there [*sic*] argument now???” (Atlanta Black Star 2016).

At the same time, tendencies toward “pragmatic reductionism” may cause individuals and institutions to privilege epigenetic data over narratives, ethnographies, historical accounts, and other forms of qualitative knowledge. Many Facebook commenters expressed that epigenetic evidence of oppression of Black people is superfluous to the sincere, compelling stories of their parents and grandparents (Atlanta Black Star 2016). As social epigenetics research progresses forward at its current pace, researchers will need to temper findings with honest disclosure of the limitations of epigenetics technologies and contextualize their results with complementary qualitative analyses. The framework of epigenetic citizenship may help researchers consider the implications of their work in person-centered ways.

## Conclusion

Social epigenetics research has room for growth. Scientists remain unsure about the durability of epigenetic marks that result from environmental insult and these may vary by exposure (Richmond et al. 2015; Simpkin et al. 2015; Zaimi et al. 2018). Furthermore, all human studies rely on associations between exposures and epigenetic changes and causality has not yet been established. Additional work using longitudinal cohort studies or intervention studies might fill this lacuna.

Despite its current shortcomings, the implications of social epigenetics are profound. The molecularization of social suffering—events like trauma, social deprivation, and racism—threatens to isolate adversity from its subjective, social, cultural, political, and historical context. The attachment of social suffering to heritable epigenetic marks may promote essentialist interpretations that may be used to justify oppression; this is especially concerning in a moment of online radicalization of young, White men and the increasing visibility of neo-Nazism, or the “alt-right” (Wilkinson 2016). Researchers engaged in social epigenetics work should thereby take great care in study design, analysis, and dissemination of results.

In this article, I engaged my experience designing and initiating a bioethnographic investigation of intergenerational trauma among Latin American migrants to problematize reductive and deterministic renderings of health inequalities experienced by structurally oppressed populations. I proposed the framework of epigenetic citizenship to contemplate the moral frontiers of social epigenetics and consider case-based scenarios in which epigenetic data can be deployed to shape health behavior, self-identity, biopolitical claims, and state control. In addition to highlighting the growing responsibilities of scientists and funders whose work entwines pliable, molecular bodies with their material, social, and political surroundings—including developing more comprehensive and theory-informed measures of sociostructural environments, engaging with larger and more diverse populations, blending ethnography with quantitative analyses, and advancing more cautious interpretations of findings—epigenetic citizenship also underscores opportunities for laypeople and policymakers to both seek and enact reparations for enduring historical harms.

## Footnotes

1 The project informing this article ultimately shifted to predominantly virtual, qualitative methods due to restrictions on in-person biomarker collection amid the COVID-19 pandemic.

2 The comments presented derive from a non-systematic content analysis of these comments. I chose to review these comments given the ‘viral’ status of the video (i.e., high view and share counts) and its integration of scholarly and popular renderings of transgenerational epigenetics. The comments presented serve as representative examples and as entry points into the arguments advanced by this paper.

3 For non-array bisulfite sequencing, absolute methylation levels are inferred from the frequency of converted cytosine and thymine residues that align to each thymine in the genomic DNA. Most often, the number of observed cytosines is divided by the total number of cytosines and thymines to yield the methylation level; however, adjustments can be made to improve accuracy, including local sequence realignment, analysis of sequence quality scores, and statistical modeling of allele distributions (Bock 2012).

4 Only the gametes that sustain harmful exposure and develop into an embryo will carry the epigenetic marks that may be seen in a subsequent generation. Given that sperm regenerate every three months, whereas female fetuses contain their lifetime supply of ova, the critical window of exposure in women is much broader than in men.
